# Access to healthcare and alternative health-seeking strategies among undocumented migrants in Denmark

**DOI:** 10.1186/1471-2458-11-560

**Published:** 2011-07-13

**Authors:** Dan Biswas, Maria Kristiansen, Allan Krasnik, Marie Norredam

**Affiliations:** 1 Danish Research Centre for Migration, Ethnicity and Health, Department of Public Health, University of Copenhagen, Øster Farimagsgade 5A, DK-1014 Copenhagen, Denmark

## Abstract

**Background:**

As in many European countries, undocumented migrants in Denmark have restricted access to healthcare. The aim of this study is to describe and analyse undocumented migrants' experiences of access to healthcare, use of alternative health-seeking strategies; and ER nurses' experiences in encounters with undocumented migrants.

**Methods:**

Qualitative design using semi-structured interviews and observations. The participants included ten undocumented South Asian migrants and eight ER nurses.

**Results:**

Undocumented migrants reported difficulties accessing healthcare. The barriers to healthcare were: limited medical rights, arbitrariness in healthcare professionals' attitudes, fear of being reported to the police, poor language skills, lack of network with Danish citizens, lack of knowledge about the healthcare system and lack of knowledge about informal networks of healthcare professionals. These barriers induced alternative health-seeking strategies, such as self-medication, contacting doctors in home countries and borrowing health insurance cards from Danish citizens. ER nurses expressed willingness to treat all patients regardless of their migratory status, but also reported challenges in the encounters with undocumented migrants. The challenges for ER nurses were: language barriers, issues of false identification, insecurities about the correct standard procedures and not always being able to provide appropriate care.

**Conclusions:**

Undocumented migrants face formal and informal barriers to the Danish healthcare system, which lead to alternative health-seeking strategies that may have adverse effects on their health. This study shows the need for policies and guidelines, which in accordance with international human rights law, ensure access to healthcare for undocumented migrants and give clarity to healthcare professionals.

## Background

**Figure 1:**
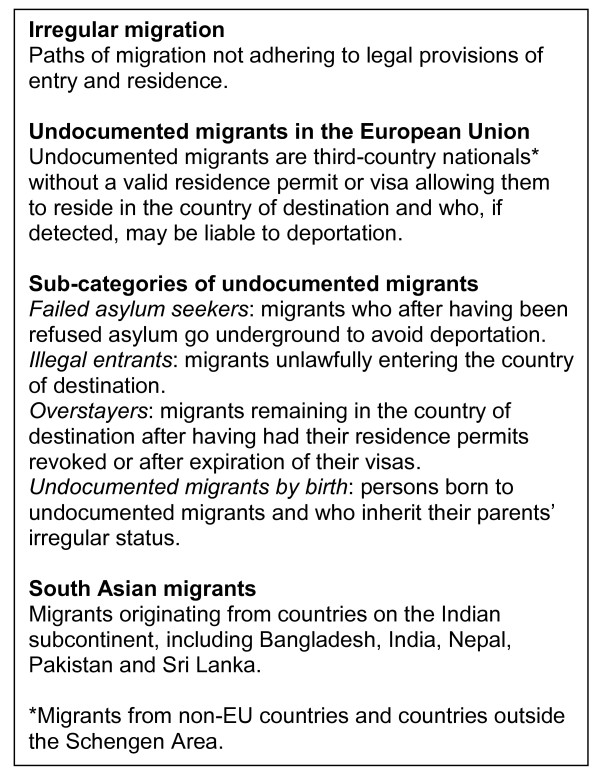
**Definitions of irregular migration, undocumented migrants in the European Union and sub-categories of undocumented migrants**.

In recent years there has been increasing focus on the flow of irregular migration to Europe [1]. It is estimated that there are between 1.9-3.8 million undocumented migrants in the European Union (EU), corresponding to 7-13 percent of the foreign resident population in 2008 [2,3]. Undocumented migrants enter Europe through both legal and illegal channels and typically find employment within the informal economy, where they serve as a low-cost and flexible labour force [1,4,5]. Figure 1 gives definitions of irregular migration, undocumented migrants in the EU and sub-categories, as used in the framework of this study [1,2,6,7]. As shown, the heterogeneous group of undocumented migrants can be divided into three sub-categories based on their original immigration status. Countries that follow the principle of jus sanguinis rather than jus soli may have a final sub-category of persons who by birth inherit their parents' irregular migratory status. Although provisions have been made by the EU to reduce the flow of irregular migration, less attention has been paid to the rights of undocumented migrants already within the EU [4,7]. Healthcare for undocumented migrants is nationally regulated by the EU member states. Apart from emergency care, entitlements vary from: full access, pre-conditioned on affiliation, duration of stay and destitution (Spain, France, the Netherlands, Portugal); partial access, providing specific preventive and curative services (Belgium, Italy, the United Kingdom); and no access to non-emergency care (remaining EU member states) [3]. Thus, undocumented migrants have restricted access to healthcare in many EU member states, who in legal framework and practice fail to recognise this group of migrants' 'right to the enjoyment of the highest attainable standard of health' as stated by the World Health Organization and codified in the 'International Covenant on Economic, Social and Cultural Rights' (1966) [5-12]. These conditions question the general stance towards international conventions on equal access to healthcare [13]. Moreover, the lack of access to healthcare may raise public health and ethical issues of concern [10]. Studies from Germany and Sweden indicate that the circumstances arising from living with an irregular migratory status and restricted access to healthcare can be an independent risk factor for reduced physical and mental health. It was specifically found that undocumented migrants face disparities in access to maternal and infant care, emergency care, medical supplies and treatment for chronic and mental health illnesses [5,14].

In Denmark irregular migration and the situation of undocumented migrants has received little public attention, most likely because the estimated number of undocumented residents is relatively low at 1,000-5,000 individuals [2,15,16]. Research on undocumented migrants in Denmark is scarce and the specific demographics of the group are unknown [15]. The Danish healthcare system is tax financed with universal access for taxpayers; all those with the right to residency have a personal health insurance card and identification number, which must be used to obtain healthcare [17]. The existing policies and legislation on undocumented migrants' medical rights appear ambiguous and are only sporadically described by decision-making bodies. In 2003 the National Board of Health stated that undocumented migrants have a right to emergency care, while healthcare professionals are not obliged to treat non-emergency cases [18]. According to the Danish Health Act, foreigners without a residence permit in Denmark may obtain non-emergency health care if it is not reasonable to refer them to their home country. However, depending on the circumstances, the Regional Council may request payment for the services [19,20]. The Danish Aliens Act states that foreigners residing illegally in Denmark may request the Danish Immigration Service for necessary healthcare services [21,22]. Nevertheless, this is de facto not an option as the Danish Immigration Service is obliged to notify the police of the whereabouts of any known undocumented migrants [18]. Lastly, there are NGOs in Denmark that can help undocumented migrants obtain medical care through informal networks of healthcare professionals. However, these organisations are at risk of being criminalised by legislation that impedes their range of activities, as citizens by law are prohibited from aiding persons residing illegally in the country [23].

In this study we examined three points: undocumented migrants' experiences of access to the Danish healthcare system, undocumented migrants' use of alternative health-seeking strategies; and emergency room (ER) nurses' experiences in encounters with undocumented migrants. By including both undocumented migrants and ER nurses, we aimed to provide a holistic overview and address the issues of access from both the undocumented migrant and provider perspective.

## Methods

We recruited ten undocumented male migrants from Bangladesh, India and Nepal by convenience sampling. The participants included 'overstayers' and 'failed asylum seekers', and were recruited through key persons within the Nepali community in Copenhagen and from a 'Copenhagen NGO'. The key persons, who mainly are in contact with undocumented migrants of South Asian origin, played a vital role in reassuring the participants that there would be no repercussions resulting from participation in the study. The key persons arranged pre-interview meetings between the researcher and the participants, before a lengthy period of fieldwork was undertaken within the community of undocumented migrants in order to gain the trust of the participants. Participants were observed in their homes and working environment and the fieldnotes were used to design the interview guide and contextualise the findings. We then recruited four ER head nurses and four ER nurses, each with a minimum of four years working experience, from four hospitals in the Capital Region of Denmark. We interviewed the head nurse at each hospital and another nurse whom the head nurse had recruited by snowball sampling. The ER setting was chosen as it is the most likely point of access to the healthcare system for undocumented migrants, with ER nurses being those most likely to be present at the initial medical encounter.

### Data generation

**Figure 2:**
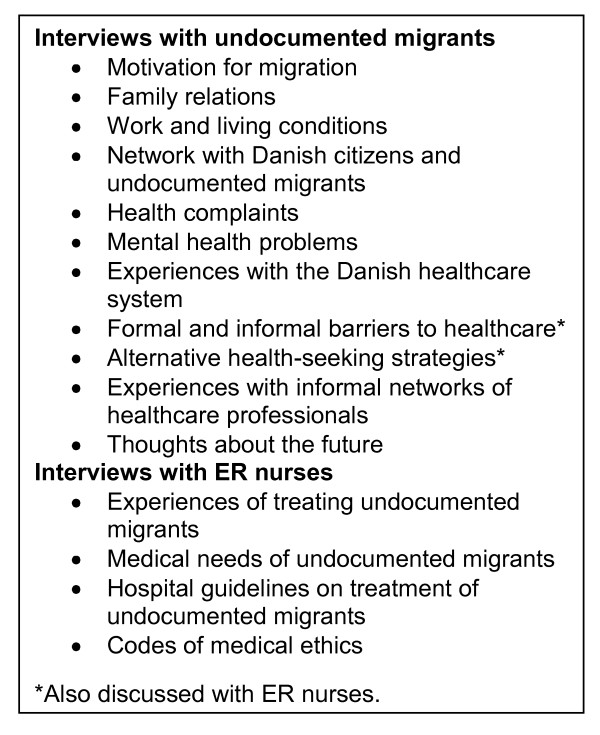
**Topics discussed in interviews**.

Data were collected from February 2009 to May 2010. DB, a researcher of Bengali-Indian origin, interviewed the participants at their homes, workplaces or at the 'Copenhagen NGO' in the language of their choice if neither Danish nor English was applicable. Eight participants were interviewed in Danish (ER nurses), eight in English (undocumented migrants) and two in Bengali (undocumented migrants). After receiving verbal consent from the participants, all interviews, except two, were audio recorded. Semi-structured interviews were used as a method of obtaining data; allowing the thoughts and experiences of the participant to emerge in an interactive process with the interviewer. The mean duration of the interviews was 45 minutes for undocumented migrants and 20 minutes for ER nurses. Figure 2 shows the topics discussed in the interviews.

### Data analysis

Data were analysed according to the three study aims. The interviews with undocumented migrants were fully transcribed, and those with ER nurses were partially transcribed, covering sequences regarding the key questions. Data were analysed according to Malteruds principles for systematic text condensation and guidelines for qualitative research. Firstly, overall impressions were obtained through multiple readings of the texts. Secondly, units of meaning were identified and coded. Thirdly, information within coded groups was condensed and abstracted. Lastly, the contents of codes were summarised into main findings [24,25]. The data material was carefully examined to compare different perspectives and viewpoints. To ensure reliability, the data analysis was independently conducted by DB and MK and opposing views were discussed with MN.

### Ethical considerations

Research on irregular migration and undocumented migrants is a highly sensitive issue that raises ethical concerns [26]. The authors regularly discussed the ethical implications of the study objectives, design, data collection, analysis and data reporting. Owing to the emotionally difficult topics discussed with the undocumented migrants, it was decided that the key person who arranged the interviews would be available for a post-interview debriefing, if the participant so wished. In the reporting data all participants have been anonymised. Before the interviews, participants were given a verbal explanation of the study as well as an information sheet in Bengali, Danish or English which explained the study including terms of anonymity, safe storage of data and the right to withdraw from the study. Due to the sensitive nature of the study, participants were required to give only verbal consent to participate. No approval from ethical committees is needed for qualitative studies according to official Danish research guidelines [27,28]. However, the study is in compliance with ethical principles for medical research as presented in the Helsinki declaration.

## Results

### Characteristics of undocumented migrant participants and their health complaints

The undocumented migrant participants had a mean age of 30 years for 'overstayers' and 38 years for 'failed asylum seekers'. The group of 'overstayers' had been in Denmark as undocumented migrants for a mean duration of 1.5 years compared with 4.5 years for the group of 'failed asylum seekers'. Table 1 gives an overview of the characteristics of the undocumented migrant participants, including health complaints and received treatments while undocumented in Denmark.

**Table 1:** Characteristics of undocumented migrant participants

Participants	Nationality	Sex	Age category	Migratory status	Health complaints	Received treatments while undocumented migrant in Denmark
P01	Bangladeshi	Male	31-40	Failed asylum seeker	Dental problems, mental health problems	None

P02	Bangladeshi	Male	31-40	Failed asylum seeker	Superficial wounds after assault, asthma	Prescription for medication from general practitioner

P03	Bangladeshi	Male	21-30	Failed asylum seeker	Psoriasis, mental health problems	Treatment for psoriasis through informal networks of healthcare professionals and at a hospital dermatology department

P04	Bangladeshi	Male	41-50	Failed asylum seeker	Leg fracture, mental health problems, cancer suspicion	Treatment for a leg fracture at hospital ER, check-up at hospital oncology department

P05	Indian	Male	41-50	Failed asylum seeker	Diabetes, high blood pressure, heart disease, mental health problems	Treatment for depression through informal networks of healthcare professionals. Medical supplies through personal networks

P06	Indian	Male	31-40	Overstayer	Dental problems, ulcer, sprained foot, mental health problems	Treatment for a sprained foot at hospital ER

P07	Nepali	Male	21-30	Overstayer	None	None

P08	Nepali	Male	31-40	Overstayer	Dental problems, mental health problems	None

P09	Nepali	Male	21-30	Overstayer	Sprained hand, skin allergy	None
						

P10	Nepali	Male	21-30	Overstayer	Mental health problems	None

Participants reported a variety of acute and chronic illnesses and health needs while living as undocumented migrants in Denmark. Mental health issues were predominant and the majority of undocumented migrant participants reported that the conditions of living with an irregular migratory status induced mental health problems, including generalised stress, anxiety and depression:

*"You don't even have your identity. You've lost your home, you've lost your relatives, you've lost your mother tongue, you've lost your culture and friends ... so if you speak about stress, this is also stress. If you think about pain ... what is pain? I cannot explain what pain is." *(Participant 5)

Participants also experienced stress from the insecurity of not having regular employment, not knowing what do to in the case of severe illness or if caught by the police as well as pressure from relatives in home countries who expected regular supplies of money.

### Undocumented migrants' experiences of access to the Danish healthcare system and alternative health-seeking strategies

Four undocumented migrants had experienced accessing the Danish healthcare system for a variety of health complaints. In all cases they were accompanied by Danish citizens, whom they mentioned as a key factor in obtaining healthcare. In the following quote, the participant describes an episode where he went to a Danish friend's general practitioner and obtained a prescription for asthma medication:

*"No, that was not a problem [to obtain treatment], because one of my friends was with me. It was not a problem, by a coincidence it was his personal doctor." *(Participant 2)

Participant 2 explained how they told the general practitioner that he was a tourist who therefore did not have a health insurance card, and thereby obtained a medical examination.

In the following quote, a Bangladeshi participant describes an episode where he received treatment at a hospital emergency room after he had an accident at work, where an industrial food mixer had fallen and fractured his leg:

*"[my Danish friend] did all the talking with the doctor, then I got the bandage on and I got crutches ... These doctors and nurses are not only human beings, they are like Gods. Within five minutes I was taken in and then X-rayed. The doctor gave me all the instructions I needed: that I had to stay in bed for 14 days and then it would be all right." *(Participant 4)

Undocumented migrants who obtained treatment expressed deep gratitude towards those healthcare professionals who would treat them despite their irregular migratory status. Participant 4, who does not speak Danish and only poor English, emphasised the necessity of the Danish friend who played a central role as a mediator in both explaining needs and negotiating the terms of care.

#### Factors influencing access to healthcare

Some undocumented migrant participants expressed uncertainty about their possibilities of obtaining healthcare in Denmark, including the right to emergency treatment, while others knew of ways of obtaining treatment. The following shows two participants' replies when questioned about the possibilities of medical treatment for undocumented migrants in Denmark:

*"After I'm out of the [asylum] system, where can I go? It's not possible in Denmark to go to a doctor or hospital." *(Participant 1)

This participant also said that undocumented migrants have no right to healthcare in Denmark and they would therefore not seek any treatment within the Danish healthcare system. Moreover, this participant did not know of any informal networks of healthcare professionals from which he could seek treatment; he would rely solely on staff from the 'Copenhagen NGO'.

Another participant said that it was not possible for undocumented migrants to access the Danish healthcare system but, contrary to Participant 1, he knew of informal networks of doctors which he had made use of once:

*"No way, not when you are blacklisted, when you are underground you cannot, but I know that some organisations are working." *(Participant 5)

This participant considered himself a 'fortunate person' because he knew of informal networks of doctors, which he said might not be the case for other undocumented migrants.

We found that fear of being reported to the police was the primary barrier to undocumented migrants seeking healthcare:

*"If I go to the doctor and the doctor is a very good Danish person, a good citizen, then maybe he will call the police. And then I would be handed over to the police and then I would have a great problem. Then my life is risky." *(Participant 5)

This quote reveals a contradiction in how migrants view 'good Danish citizens', who on the one hand are 'good' if they uphold their civic duty by not aiding undocumented migrants and reporting irregularities to the authorities, but on the other hand are viewed as 'good' if they defy these duties and do provide care.

The perceived severity and urgency of the illness condition appears to be a decisive factor as to whether undocumented migrants choose to seek medical attention:

*"I think that if they [healthcare professionals] think it [my health problem] is not so serious, then maybe they will contact the police because I have no ID card and then I will get into more trouble." *(Participant 4)

This participant was under the impression that the likelihood of receiving care was proportional to the severity of the condition, and that healthcare professionals take this into account when considering whether to provide care. In the following quote, another participant replies as to whether he would seek medical attention for conditions that are not urgent:

*"Normal problem you mean? Like fever or something else ... You don't bother to go to a doctor. You just buy some panodil [paracetamol] from the supermarket and then eat it ... Only thing is if they [undocumented migrants] don't have anything to do [any other option] they need to go to a doctor and that is the doctors' decision what they are going to call police or not. That is totally up to them actually." *(Participant 2)

The undocumented migrant participants underlined that any contact with the authorities could ultimately result in deportation. Accordingly participants were wary of situations that could compromise their safety, for example, they would carefully consider whether any illness was sufficiently severe to warrant medical attention. Likewise participants stated that they were reluctant to seek attention for conditions which they in their home countries normally would not hesitate to be examined for.

Another barrier may be arbitrariness in healthcare professionals' attitudes towards treatment of undocumented migrants. This is illustrated by a participant who contacted a dermatology department to enquire about the possibilities of medical treatment for psoriasis:

*"He was quite an important doctor. He said: If you don't show the card then you do not get a file. He said: Okay, I can treat you today, but next [time] the doctor will change, and he may not be willing to treat you." *(Participant 3)

When such experiences are spread around within the undocumented migrant community, it contributes to the perception that visits to the healthcare system may not always result in treatment, and further emphasises the need for alternative health-seeking strategies.

One participant described how language and cultural differences may place undocumented migrants in a more vulnerable position and obstruct access to healthcare:

*"Most of the illegal immigrants can't speak the language [Danish] and not even English. They need to have some person with them who can explain about them or else they will be in a big trap down there. Language miscommunication; maybe the doctor wants to ask something and he can't answer and everything will be ... It will not work in that way." *(Participant 2)

In addition language and cultural differences may be connected to low health literacy, where the lack of ability to navigate and negotiate in a complex healthcare system may construct barriers of access for undocumented migrants.

Thus, the participants reported different factors influencing access to healthcare and affecting how they responded to illness and health needs.

#### Alternative health-seeking strategies

Problems in accessing healthcare may give rise to alternative health-seeking strategies.

The majority of undocumented migrant participants recounted situations where they had postponed or avoided contact with healthcare professionals and instead had used alternative treatment methods:

*"They [undocumented migrants] are just their own doctors. They just take the medicine. Perhaps they have just called Bangladesh or their own country and have called their doctor or their parents and have explained the problem to that doctor, and perhaps that doctor told them that you should take this kind of medicine." *(Participant 2)

Participants said that it was common for undocumented migrants to contact doctors in their home countries for medical advice and for persons in their network to bring medicine from their home countries. None of the participants expressed concern in regard to receiving long-distance examinations, but viewed it as safe and easy way to gain medical advice.

Participant 5, who had high blood pressure and a heart disease, explained how fear prevented him from entering the healthcare system for a check-up and how instead he made use of an old prescription for heart medicine:

*"I need regular treatment, but I did not have regular treatment. So I used the prescription from when I was an asylum seeker, which is not so good for the health, because you know, the prescription has to change [...] So from the beginning, if I get the same medicine for a long time it is like a poison. So in that way I was taking poison, but I did not have any other way." *(Participant 5)

This participant was able to procure the necessary medicine through persons in his network who had contact with heart patients with extra tablets or buy them on the illegal drug marked in Copenhagen. He was fully aware that it could be potentially harmful to use an old prescription without receiving any medical follow-up or adjustments, but yet he did not see any other options.

In the following quote, the participant recounted how he had noticed a growth and had suspected it to be cancer but waited eight months before finding a way to get an examination:

*"I had kind of a growth here. It took eight months for me to get in. Some experts looked at me. I was afraid that it was cancer, but the doctor said it is not cancer." *(Participant 4)

Likewise, other undocumented migrant participants reported being aware of all symptoms, even minor symptoms. They emphasised the importance of good self-care when healthcare is not easily available. This quote also illustrates the stress which undocumented migrants may experience when not being able to obtain certainty about medical conditions. Again, in this case, the delay of treatment was influenced by this participants' inability to procure a Danish citizen that could help him seek medical attention.

### ER nurses' experiences in encounters with undocumented migrants

ER nurses from three out of four hospitals had either themselves or knew of colleagues who had provided healthcare for undocumented migrants or for persons whom they suspected were undocumented migrants. The nurses reported that undocumented migrants seldom appeared in the ER; nevertheless, they could be characterised by a treatment-seeking pattern that differed from that of most other patients. It was found that undocumented migrants sometimes delayed treatment and that they appeared insecure and preferred quick treatment to being hospitalised:

*"A couple of months ago a person [undocumented migrant] came in with a really nasty, infected wound. I am guessing that it was from a dog bite. It was on the right thigh and it was badly infected. He was reddish and had a fever, so we recommended that he was hospitalised and treated with intravenous antibiotics. But he didn't want that. So in the end we patched up his wounds and then sent him away with a prescription for penicillin." *(ER nurse 2)

All interviewed ER nurses expressed that nurses have a duty to treat all patients in need of medical care regardless of whether the patient is an undocumented migrant. Thus it was underlined that non-discriminatory care and patient confidentiality is a matter of professional integrity:

*"Those who come here have the right to be treated. And whether they are here legally or not, that must be up to some other authorities to decide. That is not up to us to decide." *(ER nurse 5)

Similarly, another ER nurse said:

*"The moment I put on my nurse's coat I am a nurse and have a duty to treat whoever comes." *(ER nurse 7)

However, arbitrariness in healthcare professionals' attitudes towards treatment of undocumented migrants was described in one interview where the nurse recounted an episode where the police were contacted:

*"This colleague of mine, I don't know if she was afraid or felt insecure or just thought that he [undocumented migrant] shouldn't be allowed to be here. In any case she called the police and they came. And the end of it was that he got the medical treatment and was then subsequently arrested and taken away." *(ER nurse 2)

This episode gave rise to internal discussions among the nurses about whether it was acceptable that the police had been contacted. ER nurse 2 described how opposing opinions clashed in later discussions between the nurses and how the matter remained unresolved, which she imputed to the lack of internal guidelines:

*"Our heads of departments have not issued any common guidelines on how to act in such a situation. But I also think the problem is that they have just as much difficulty in giving out any common guidelines, because you can say that it is an area where legally we stand very weak. And it is because, according to the law, that somewhere we have an obligation to contact the police and say: well, this is a violation of the law. And then we have the oath of confidentiality which does not permit us to contact the police, and then you can discuss what weighs heaviest." *(ER nurse 2)

This quote shows the contradiction between the conception of being loyal to law, implying that it is a civic duty to notify the authorities of persons residing illegally in the country, and then the professional obligation to always act in the patients' best interest. This nurse touched upon the discrepancy between the law and codes of medical ethics which may create uncertainty about their position when encountering undocumented migrants. Furthermore, these uncertainties are amplified by nonexistent policies and guidelines within the organisation, which may make it difficult for the healthcare professionals to navigate.

#### Challenges for ER nurses in treating undocumented migrants

ER nurses reported possible challenges in treating undocumented migrants, including language barriers and undocumented migrants' lack of trust in the healthcare system, which may cause them to be less informative about their situation and illness. One nurse mentioned insecurities regarding the correct standard procedures and expressed a need for guidelines on how to respond to undocumented migrants, while the remaining nurses expressed that they considered guidelines were already implicitly included in codes of medical ethics. Another challenge concerned undocumented migrants who in an attempt to obtain healthcare professed a false identity by using another person's health insurance card:

*"If you are scanned for pain in the abdomen and the person you have borrowed the health insurance card from has had his appendix removed, then we might say to ourselves: well, then it is not appendicitis and then you could die from a perforated appendix. So, of course it is dangerous. But it is also really, really foolish ... then it is better to say that you do not have any [health insurance card]." *(ER nurse 3)

The nurses explained that this practice may ultimately have fatal consequences, and they underlined the importance of explaining to those patients that they could be treated anonymously.

## Discussion

In this study we found that undocumented migrants face formal and informal barriers in accessing the Danish healthcare system. These barriers include limited medical rights, arbitrariness in healthcare professionals' attitudes, fear of being reported to the police, poor language skills, lack of network with Danish citizens, lack of knowledge about the healthcare system and lack of knowledge about informal networks of healthcare professionals. These barriers induced alternative health-seeking strategies including: self-medication, contacting doctors in home countries and borrowing health insurance cards from Danish citizens. ER nurse participants expressed willingness to treat all patients regardless of their migratory status but also reported insecurities about the correct standard procedures. Other challenges for ER nurses included language barriers, issues of false identification and not always being able to provide appropriate care.

### Strengths and limitations of the study

Research is scarce on undocumented migrants' health needs, health behaviour and access to healthcare in Europe. Overall, this is due to the apprehensive and clandestine nature of undocumented migrants, making research both complex and challenging. Strong contacts in the relevant environment and a similar ethnic background of the interviewing researcher and the undocumented migrant participants along with lengthy pre-interview fieldwork enhanced the level of interpersonal trust and enabled us to conduct the interviews. In relation to the recruitment of ER nurses, the snowball sampling may be a possible cause of bias if the head nurses only selected nurses with similar positive attitudes towards the treatment of undocumented migrants, which would result in unanimous findings. Moreover, when conducting research on politically and ethically sensitive topics, there is a risk of social desirability bias as participants may withhold information that can bring discredit to themselves, the group they represent, or their profession. In the present study this was further amplified as diversity in attitudes towards undocumented migrants' access to healthcare may have been combined with insecurity or fear regarding the possible implications if participants had been identified. However, it was our impression that both nurses and migrants freely engaged in the interviews, and we made every possible effort to secure anonymity and provide a comfortable interview setting throughout the study.

The study population consisted of a relatively small, heterogeneous group of undocumented South Asian migrants and a small group of ER nurses. The findings are clearly influenced by individual and contextual characteristics related to for instance, age, gender, ethnicity, undocumented migrant sub-category, and extent of experience encountering undocumented migrants. However, the themes regarding formal and informal barriers to accessing healthcare may be relevant to other groups of undocumented migrants and healthcare professionals. Thus, it is expected that these themes may be shared by other groups of undocumented migrants who experience similar insecure life circumstances. Moreover, despite varying national policies and healthcare systems, undocumented migrants have restricted access to healthcare in many countries making the findings on alternative health-seeking strategies and challenges for healthcare professionals applicable beyond a Danish context.

### The results in perspective

The results of this study are supported by existing literature demonstrating that there are problems of access to healthcare for undocumented migrants in most European countries [3,5-7,14]. The main formal barrier identified in this study were restrictions on medical rights as undocumented migrants' only formal option for non-emergency care is through the Danish Immigration Service which de facto is not an option, leaving limited possibilities for healthcare and revealing discrepancies between entitlements and actual access.

Previous studies have shown how undocumented migrants respond to formal and informal barriers by practising self-medication, and thus delaying treatment [5,29]. These alternative health-seeking strategies give rise to concern as self-medication and delayed treatment may lead to harm and aggravated health conditions. This was seen in the case of Participant 5, who had been using the same prescription for his heart disease for several years without receiving any medical follow-up or adjustments. In a German study, NGO physicians reported that undocumented migrants had a higher degree of illness on average than did patients in normal practice because they delayed treatment. Instead undocumented migrants had a wider use of non-prescription medications and illegally obtained medications which may be of public health concern as unregulated use of medications such as antibiotics can increase resistant strains [5].

We found that undocumented migrants primarily operate on the basis of fear, which also induces the use of alternative healthcare-strategies, as contact with the healthcare system may be connected to fear of being denied care or being reported to the police. Accordingly in a study among undocumented migrants in Sweden, 67 percent of the respondents reported that that they felt their risk of being arrested by authorities at a hospital was either 'quite high' or 'extremely high' [14].

An American study found, that undocumented Latino migrants who expressed fear about seeking healthcare were likelier to report difficulties obtaining prescribed medication, medical and dental healthcare [30].

The interviews with ER nurses gave insight into policy and practice concerning access to healthcare for undocumented migrants, thereby shedding light on how encounters with undocumented migrants are perceived within an organisational context. This further complemented and contextualised the findings from the interviews with undocumented migrants. From the perspective of professionals situated within the healthcare system, our results show that most ER nurses are willing to treat undocumented migrants as they would treat any other patient, without taking their migratory status into consideration. However, one nurse reported a case in which the police had been contacted. This case confirms that insecurities may arise from the lack of guidelines on how to respond to undocumented migrants seeking healthcare. All nurses reported that there are no guidelines on undocumented migrants' right to emergency care other than those codified in general codes of medical ethics [31]. Thus, due to the lack of guidelines, undocumented migrants' access to emergency care depends on how ER nurses interpret and act on codes of medical ethics and personal convictions. It has been argued that healthcare professionals' ethical obligation of confidentiality should extend to undocumented migrants thereby upholding their duty to do no harm [32]. However, it has been previously reported that healthcare professionals may experience clashes between what their medical ethics tell them to do and the common incriminatory discourse regarding undocumented migrants [6]. While this study has focused on ER nurses, other reports have shown that undocumented migrants may face access problems earlier in the system, for example, when engaging with hospital administrators, who are not bound by the same codes of medical ethics [6]. This shows that there may be barriers on different levels within the healthcare system underlining the complexity of undocumented migrants' access to healthcare.

Discussing undocumented migrants' access to healthcare is relevant in a human rights framework. The fundamental principles of non-discrimination and 'the right to health' are outlined in international human rights laws [9,12,18]. These are, inter alia, codified in the 'International Covenant on Economic, Social and Cultural Rights', and are further elaborated in the 'General Comment No. 14' (2000), where it is specified that states are under the obligation to respect an individual's right to health by refraining from denying or limiting equal access for all persons, including undocumented migrants, to preventive, curative and palliative care [9,33]. Thus, states should promote, provide and facilitate measures to realise this [12]. In reference to international human rights law, it can therefore be argued that immigration status should not influence entitlements to healthcare, and that a first step towards securing undocumented migrants medical rights would be to separate the intertwined relationship between immigration control and healthcare [13].

Romero-Ortuño describes two approaches to the provision of healthcare for undocumented migrants: 'the humanitarian approach' which is based on human rights principles, and 'the utilitarian approach' which argues that care should be provided in order to protect the host population from untreated communicable diseases [34]. In relation to our findings on the lack of access to non-emergency care, other studies have argued that preventive and early care is likely to be more efficient and economically prudent than treatment at a late stage [12,34]. The economic aspects are often decisive when undocumented migrants' entitlements are negotiated. With reference to existing experiences, Romero-Ortuño argue that it is feasible for European healthcare systems to assume the health needs of undocumented migrants without undergoing major changes to the funding or organisation [34].

### Implications for research, healthcare policy and practice

Our findings have implications for research, healthcare policy and practice. Firstly, health needs, use of healthcare services and health-seeking strategies are intertwined and may evolve over time as contextual factors change. Changes in legislation and policies are important factors in framing undocumented migrants' access to healthcare, just as these factors influence healthcare professionals practice. To explore this dynamic and complex contextual relationship, future studies should use a longitudinal design with multiple interviews, preferably combining interviews and observations among different groups of undocumented migrants in order to explore possible differences in needs and health-seeking strategies. Moreover, future studies should include healthcare professionals situated in different sections and levels of the healthcare system.

Secondly, there is a need for development and implementation of uniform policies and guidelines ensuring access to healthcare for undocumented migrants and which, furthermore, are publicised and disseminated to healthcare professionals, hospital administrators and undocumented migrants.

Thirdly, in many European countries there are parallel healthcare systems where NGOs and informal networks of healthcare professionals have taken over the role of the public healthcare system in providing care for undocumented migrants [29]. However, parallel systems may give rise to concern as they seldom have the same facilities as regular hospitals, which may result in difficulties in providing an appropriate quality of care. Moreover, the establishment of parallel systems may risk legitimising the lack of action on the part of the welfare state. Therefore with reference to international human rights law, we argue that parallel systems should only be a temporary solution as welfare states have the responsibility of providing healthcare for undocumented migrants.

## Conclusions

Undocumented migrants reported difficulties accessing healthcare in Denmark due to both formal and informal barriers. We found that encountering such barriers induce alternative health-seeking strategies that may have adverse effects on their health. ER nurses expressed willingness to treat all patients regardless of their migratory status, but also reported challenges in the encounters with undocumented migrants. The existing policies and legislation on undocumented migrants' medical rights appear ambiguous and are in practice only inadequately implemented. Moreover the lack of guidelines within the healthcare system may give rise to insecurities about how healthcare professionals should respond to undocumented migrants. This study shows the need for policies and guidelines, which in accordance with international human rights law, ensure access to healthcare for undocumented migrants and give clarity to healthcare professionals.

## Competing interests

The authors declare that they have no competing interests.

## Authors' contributions

DB participated in the design, collected the data, performed analyses, interpretation of data and drafted the article. MK participated in the design, performed analyses, interpretation of data and revised the article. AK participated in the interpretation of data and revised the article. MN participated in the design, interpretation of data and revised the article. All authors read and approved the final manuscript.

## Pre-publication history

The pre-publication history for this paper can be accessed here:

http://www.biomedcentral.com/1471-2458/11/560/prepub
